# Optical Coherence Tomography Angiography Findings in Stargardt Disease

**DOI:** 10.1371/journal.pone.0170343

**Published:** 2017-02-02

**Authors:** Rodolfo Mastropasqua, Lisa Toto, Enrico Borrelli, Luca Di Antonio, Peter A. Mattei, Alfonso Senatore, Marta Di Nicola, Cesare Mariotti

**Affiliations:** 1 Moorfields Eye Hospital, NHS Foundation Trust, London, United Kingdom; 2 Department of Medicine and Science of Ageing, Ophthalmology Clinic, University G. D'Annunzio Chieti-Pescara, Chieti, Italy; 3 Department of Experimental and Clinical Sciences, Laboratory of Biostatistics, University "G. d'Annunzio" Chieti-Pescara, Chieti, Italy; 4 Department of Surgical and biomedical Science, Ophthalmology Clinic, University of Marche, Ancona, Italy; Medical University of South Carolina, UNITED STATES

## Abstract

**Background:**

to assess vessel density of superficial capillary plexus (SCP), deep capillary plexus (DCP) and choriocapillaris (CC) in advanced Stargardt disease (STGD) using optical coherence tomography angiography (OCTA) and correlate these findings with macular function using pattern electroretinogram (PERG) and multifocal electroretinogram (mfERG).

**Methods:**

Twelve patients (24 eyes) with advanced STGD underwent vessel densities and macular thickness measurements using OCTA. A control group of 24 healthy controls (24 eyes) was chosen for comparison. In the STGD group correlation between vessel density and macular thickness and between macular function and morphologic parameters were evaluated.

**Results:**

Whole parafoveal vessel density (VD) of SCP was significantly lower in STGD group compared to the control group (p<0.05). Foveal VD and whole parafoveal VD of the DCP were significantly lower in STGD group compared to the controls (p<0.05). CC was significantly decreased in STGD compared to controls (p<0.05). Foveal macular thickness (MT), full parafoveal MT, and inner limiting membrane (ILM)-inner plexiform layer (IPL) parafoveal MT thickness were decreased in STGD eyes compared to controls (p<0.001). PERG and mfERG were both significantly reduced in STGD compared to controls (p<0.001). A direct correlation was found between full parafoveal MT and vessel density in the STGD group.

**Conclusions:**

Patients with advanced STGD showed a reduction of SCP, DCP and CC compared to healthy eyes related to a reduction of total and ILM-IPL macular thickness. These results suggest that both retinal capillaris plexuses and choriocapillaris reduction occur in STDG along with inner and outer retinal thinning.

## Introduction

Stargardt disease (STGD), also known as Stargardt macular dystrophy, is one of the most prevalent inherited maculopathy, caused by mutations in the ABCA4 gene [1,2]. It is characterized by a progressive bilateral loss of central vision that can present from early childhood to late adulthood and includes a wide range of clinical and electrophysiological findings [3–5]. STGD is characterized by photoreceptor and choriocapillaris loss, with abnormal lipofuscin (LF) accumulation in a wide variety of patterns [2,6–9].

Some recent studies using either optical coherence tomography (OCT) or fluorescein angiography (FA) and indocyanine green angiography (ICG) demonstrated choriocapillaris atrophy in eyes with STGD [10–12]. Choriocapillaris atrophy is a direct consequence of retinal pigment epithelium (RPE) atrophy suggesting a role of RPE in modulation of choriocapillaris structure and function. RPE, in fact, produces VEGF and VEGF receptors expressed on the choroidal endothelium facing the RPE [13]. In turn choroidal vessels provide the vascular support to outer retinal layers suggesting a possible role of choriocapillaris atrophy in photoreceptors degeneration [11]. This suggests that vascular involvement is probably plays an important pathogenetic role in STGD.

OCT angiography (OCTA) has recently been developed to study retinal and choroidal microvasculature without dye injection and has already been used to study the superficial and the deep retinal vascular plexuses and choriocapillaris in several retinal vascular diseases [14–18].

The aim of this study was to assess vessel density of superficial capillary plexus (SCP), deep capillary plexus (DCP) and choriocapillaris (CC) in advanced STDG using OCTA, correlating findings with macular function using electrophysiology testing and by comparing vessel density of eyes with advanced STGD and eyes of normal subjects.

## Materials and Methods

### Study participants

Twelve consecutive patients (24 eyes) with a diagnosis of advanced STGD (ascertained by biomicroscopy according to the classification proposed by Fishman et al) [19] referring to Medical Retina Service of the Ophthalmology Eye Clinic of University “G. d’Annunzio” between July 2015 and March 2016 for routine retinal check-up were enrolled for the study. This study was approved by our Institutional Review Board (Department of Medicine and Science of Aging, University "G. d'Annunzio" Chieti-Pescara, Italy), and patients provided signed informed consent for the use of their data. The study adhered to the tenets of the Declaration of Helsinki.

Criteria for inclusion were: 1) best-corrected visual acuity (BCVA) greater than or equal to 1.0 LogMAR in the study eye at baseline examination (to ensure proper execution of functional examination). The exclusion criteria were causes other than STGD capable of reducing visual acuity such as media opacities, other macular or diffuse retinal pathologies impairing visual function or retinal function at electrophysiology testing.

A control group was composed of 24 healthy age-matched patients (24 eyes) presenting to the same centre.

### Study Protocol

At baseline, all patients underwent a complete ophthalmic evaluation, including assessment of BCVA using Early Treatment Diabetic Retinopathy Study (ETDRS) charts, tonometry, slit-lamp biomicroscopy and indirect fundus ophthalmoscopy. Furthermore, all patients underwent evaluation of vessel density of SCP, DCP and CC with an XR Avanti^®^ AngioVue OCTA (Optovue Inc., Fremont, CA). Patients with STGD underwent mfERG and PERG using Retimax (CSO, Florence, Italy) to evaluate macular function.

### Procedures

#### SD-OCT angiography with XR Avanti

XR Avanti^®^ AngioVue OCTA (Optovue Inc., Fremont, CA) is a device with a high-speed of 70,000 axial scans per second, using a light source of 840 nm, and an axial resolution of 5μm. This system is based on the SSADA algorithm (Version: 2015.1.0.90) that uses blood flow as an intrinsic contrast. Flow is detected as a variation over time in the speckle pattern formed by interference of light scattered from red blood cells and adjacent tissue structure [14,20].

Before imaging, pupils were dilated with a combination of 0.5% tropicamide and 10% phenylephrine. Study participants underwent SD-OCT imaging following a protocol that included AngioVue OCT 3D volume set of 6x6 mm. An internal fixation light was used to centre the scanning area. The OCT signal position and quality were optimized using the “Auto All” function which performs in sequence the “Auto Z” to find the best position for obtaining the retina OCT image, the “Auto F” to find the best focus for the particular subject’s refraction, and the “Auto P” to find the best polarization match for the particular subject’s ocular polarization.

One FastX (horizontal raster) set and one FastY (vertical raster) set were performed for each acquisition scan. Each set takes approximately 3 seconds to complete. After the completion of the FastX and FastY sets, the software implemented motion correction technology (MCT) to remove saccades and minor loss of fixation. Scans with low quality (i.e., if the subject blinked or if motion artefacts were present in the data set) were excluded and repeated until a good quality was achieved. Three scans for each patient were captured, then the one with the best quality (without significant motion artefacts and with a signal strength index >60) was considered for analysis.

#### Vascular layer segmentation

Vascular retinal layers were visualized and segmented as previously described [21]. The superficial plexus consists of the capillaries between the inner limiting membrane (ILM) and the posterior boundary of the inner plexiform layer (IPL) (including the nerve fiber layer and the ganglion cell). The deep plexus consists of the capillaries between the posterior boundary of the IPL and the posterior boundary of the outer plexiform layer (OPL) (including inner nuclear layer). The choriocapillaris consists of capillaries in a 30μm thick layer posterior to the retinal pigment epithelium-Bruch membrane junction. Based on these default settings, the boundaries of the superficial network extended from 3 μm below the ILM to 15 μm below the IPL. The deep capillary network extended from 15 μm to 70 μm below the IPL. The choriocapillaris extended from 30 to 60 μm below the retinal pigment epithelium.

The software option to remove projection artefacts from inner vascular plexus in the outer retina was selected. If necessary, manual adjustment of layer segmentation in case of projection artefacts from inner vascular plexus in the deep vascular plexus was performed by two retina specialists (LT and EB) and the manual adjustment of segmentation was chosen only if a consensus was reached.

Two observers (LT and EB), independently checked image quality and excluded poor quality images leading that could have led to possible segmentation errors.

#### Quantitative vessel analysis

Objective quantification of vessel density was evaluated for each eye using the SSADA software. Quantitative analysis was performed on the OCTA en face images for each eye using the AngioVue software. The vessel density was defined as the percentage area occupied by vessels in a circular region of interest (ROI) centred on the centre of the foveal avascular zone with a diameter of 3x3 mm included inside the 6x6 mm scan area. The AngioVue software automatically splits the ROI into two fields: the foveal area, a central circle with a diameter of 1 mm; and the parafoveal area that constitutes the remaining part inside the ROI. The three capillary systems were evaluated with quantitative analysis in the foveal and parafoveal areas and with qualitative analysis in the whole 6x6 mm scan area.

The vessel density was calculated using the formula previously described [14,18], as follows:

$$Vessel\;density=\frac{\int V∙dA}{\int dA},$$

where V is 1 when the OCTA value is above a background threshold and 0 otherwise; A is the area of interest.

For each patient, foveal and parafoveal vessel density (VD); and parafoveal VD in different quadrants (temporal, superior, nasal, inferior) of the SCP, DCP and CC were calculated.

#### Qualitative vessel analysis

Two independent observers (LT and EB) subjectively evaluated OCTA in the 6x6 mm scan of best quality. Vascular anomalies were evaluated in terms of vessels calliper (regular or irregular), vessel coarse (regular or irregular such as distorted) and density (normal or rarefied). Perifoveal capillaries were evaluated to disclose disruption or integrity of the perifoveal anastomotic arcades.

#### Foveal and parafoveal retinal thickness analysis

Foveal macular thickness (MT), full parafoveal MT, and parafoveal MT in different retinal quadrants and ILM-IPL parafoveal MT were automatically calculated by the software on the OCTA 6x6 mm volume scan (XR Avanti^®^; Optovue, Inc., Fremont, CA, USA). A circular ROI centred on the centre of the foveal avascular zone with a diameter of 3.0 mm was considered for retinal thickness analysis: central foveal area (1 mm of diameter) and parafoveal area that constitutes the remaining part inside the ROI (full parafoveal area or temporal, superior, nasal and inferior quadrants).

#### Electrophysiology testing

Pattern electroretinograms (PERG) and multifocal ERGs (mfERG) were recorded for each patient, according to the International Society for Clinical Electrophysiology of Vision (ISCEV) protocols [22,23]. P50 and N95 amplitude and latency of the PERG test were analysed for each patient.

For mfERG, the ocular fundus was segmented by an array of 61 hexagons, and average responses for the implicit times and amplitudes of N1 (first negative component) and P1 (first positive component) of the first-order kernel were calculated for five regional ring groups (R1 to R5). Amplitude was measured from the baseline to the trough (N_1_) or peak (P_1_) of the deflection. Area 1 corresponds to the fovea, area 2 to the parafovea, and areas 3–5 correspond to the outer portions.

### Main outcome measures

The main outcome measures were foveal VD; whole parafoveal VD; and parafoveal VD in different retinal quadrants (temporal, superior, nasal and inferior) of the SCP, DCP and CC; foveal MT; full parafoveal MT; and parafoveal MT in different retinal quadrants and ILM-IPL parafoveal MT; PERG P50 and N95 amplitude and latency, mfERG P1 and N1 amplitudes for five regional ring areas; correlation between vessel density and retinal thickness and between morphologic parameters (vessel density and retinal thickness) and functional parameters (PERG P50 and N95 component and mfERG N1 P1 component in the five regional rings groups) were evaluated.

### Statistical analysis

All quantitative variables were presented as mean and standard deviation in the results and in the tables. To detect departures from normality distribution, Shapiro-Wilk’s test was performed for all variables. The statistical differences between groups were analysed by conducting one-way Analysis of Variance (ANOVA). Pearson’s correlation coefficients were estimated to evaluate the linear correlation among variables in STDG patients.

The false discovery rate correction (FDR) was used to control the family-wise type I error rate and an FDR adjusted p value less than 0.05 was determined to be statistically significant.

Statistical calculations were performed using Statistical Package for Social Sciences (version 20.0, SPSS Inc., Chicago, IL, USA).

## Results

### Patients population

Two patients (four eyes) of the STDG group were excluded from the study due to unreliable layer segmentation.

### Demographic data

The mean age was 47.6±15.6 years (range 24–66 years) for patients with STDG and 45.3±12.1 years (range 26–62 years) for healthy controls (p = 1.0). In the STGD group 58% were females and 42% patients were males and in the control group 60% were females and 40% were males (p = 1.0).

Nine out of 10 patients showed a stage III STGD and one out of 10 showed a stage II-III STDG according to Fishman classification.

### Visual acuity and electrophysiology tests

The mean BCVA values were 0.9±0.1 logMAR in the STGD group and 0.0±0.5 logMAR in the control group (p<0.001). P and N amplitude in five regional rings were significantly reduced in the STDG group compared to controls (p<0.001). In particular, foveal P1 and N1 amplitude were 0.41±0.80 and -0.17±0.63, respectively, in STDG group vs 0.88±0.04 and -0.40±0.00 in the control group. Parafoveal P1 and N1 amplitude were 0.22±0.50 and -0.30±0.57, respectively, in STDG group vs 0.61±0.01 and -0.36±0.06 in the control group (p<0.001).

Compared to the normal patients, the STDG group showed significantly reduced PERG P 50 and N95 amplitude (1.60±1.51 μV and 2.22±1.53 μV in the STDG group vs 3.39±1.32 μV and 5.25±1.70 μV in the control group) (p<0.001) and significantly delayed PERG P50 and N95 latency (56.99±6.63 msec and 116.45±14.23 msec in the STDG group vs 50.58±1.46 msec and 98.73±4.56 msec in the control group) (p<0.001and p = 0.001, respectively).

### Qualitative vessel density analysis

**Fig 1:**
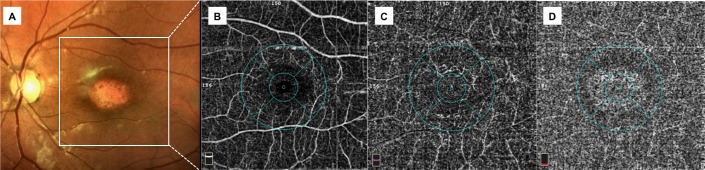
Multimodal imaging of a patient (26 years-old) with Stargardt disease at stage III (Fishman classification). A colour fundus photograph showing macular atrophy with completely resorbed flecks; B, C optical coherence tomography angiography of superficial plexus showing rarefaction of superficial retinal vessels and enlargement of the foveal avascular zone with irregular contour, deep plexus with severe vessel rarefaction and some projections of the main retinal vessels due to retinal atrophy; D optical coherence tomography angiography of choriocapillaris showing an apparent increase of choriocapillaris density centrally with a brighter aspect due to retinal pigment epithelium atrophy and some black dots possibly related to areas without perfusion.

**Fig 2:**
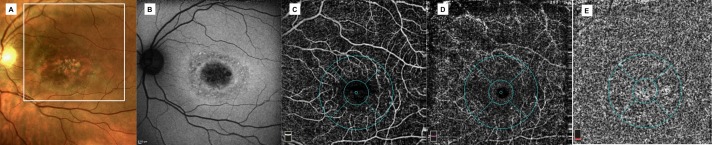
Multimodal imaging of a patient (41 years-old) with Stargardt disease at stage II-III (Fishman classification). A colour fundus photograph showing atrophic macular changes, mostly resorbed flecks in the infero-temporal extrafoveal area and some pisciform and round flecks in the remaining extrafoveal areas; B fundus autofluorescence showing central focal areas of hypoautofluorescence due to retinal pigment epithelium atrophy and some hyperautofluorescent areas due to lipofuscin accumulation related to flecks; C, D optical coherence tomography angiography of superficial plexus showing rarefaction of superficial retinal vessels and enlargement of the foveal avascular zone, deep plexus with severe vessel rarefaction and some projections of the main retinal vessels due to retinal atrophy; E optical coherence tomography angiography of choriocapillaris showing some black dots possibly related to areas without perfusion.

OCTA provided detailed images of the retinal and choriocapillaris microcirculation. In all patients (20 eyes) of the STDG group vessels were diffusely rarefied in the foveal and parafoveal area in the superficial and deep plexus (Figs 1 and 2). The calibre of both plexuses was regular with normal course. An interruption of the perifoveal anastomotic arcade was present in all cases (20 eyes) but with varying extent. In 15 out of 20 eyes (75%), the choriocapillaris showed the presence well delineated black dots possibly related to areas without perfusion.

### Quantitative vessel density analysis

Foveal VD, parafoveal FVD of the superficial, deep plexus and choriocapillaris are shown in Table 1. Parafoveal VD of SCP was significantly lower in the STGD group compared to the control group (p<0.05). Foveal VD and parafoveal VD of the DCP were significantly lower in the STGD group compared to the control group (p<0.05). The same applies for CC that was significantly decreased in STGD disease compared to normal controls (p<0.05) (Table 1). Parafoveal vessel density of different retinal quadrants of SCP, DCP and CC was significantly lower in STDG compared to controls (p<0.05).

**Table 1:** Vessel density values of superficial plexus, deep plexus and choriocapillaris.

	*Stargardt Group*		*Control group*	*p-value*
***Foveal Superficial Vessel Density (%)***		27.25±10.19	31.44±5.41	*0*.*153*
***Parafoveal Superficial Vessel Density (%)***		46.34±4.04	52.55±2.94	***<0*.*001***
***Foveal Deep Vessel Density (%)***		37.52±9.51	29.68±7.42	***0*.*015***
***Parafoveal Deep Vessel Density (%)***		47.38±4.25	59.09±2.79	***<0*.*001***
***Foveal Choriocapillaris Vessel Density (%)***		54.87±24.84	27.51±5.37	***<0*.*001***
***Parafoveal Choriocapillaris Vessel Density (%)***		60.63±6.46	67.11±1.40	***<0*.*001***

All p-value reported in bold in table are significant after FDR-adjustment with q = 0.05

### Retinal macular thickness

Foveal MT, full parafoveal MT, and ILM-IPL parafoveal MT thickness were significantly decreased in STGD eyes compared to control eyes (p<0.001) (Table 2).

**Table 2:** Foveal macular thickness, parafoveal macular thickness and inner limiting membrane-inner plexiform layer parafoveal macular thickness in the Stargardt disease group and in the control group.

	*Stargardt group*	*Control group*	*p value*
***Foveal Macular Thickness (μm)***	147.79±54.89	249.65±56.18	***<0*.*001***
***Full Parafoveal Macular Thickness (μm)***	219.07±22.87	324.53±17.40	***<0*.*001***
***Inner limiting membrane-inner plexiform layer Parafoveal Macular Thickness (μm)***	76.60±8.65	114.00±27.54	***<0*.*001***

All p-value reported in bold in table are significant after FDR-adjustment with q = 0.05

### Correlation analysis between different parameters

A direct correlation was found in STGD patients between FPMT and vessel density of SCP (R = 0.483 p-value 0.006), DCP (R = 0.645 p-value <0.001) and CC (R = 0.578 p-value 0.001). The thinnest the PMT the highest is vascular depletion of the superficial and deep plexus and of the choriocapillaris. A direct correlation was also found between ILM-IPL PMT and vessel density of SCP (R = 0.358 p-value 0.048), DCP (R = 0.467 p-value 0.008) and CC (R = 0.464 p-value 0.009).

No correlation was found between functional parameters such as PERG and mfERG and both vessel density and macular thickness. Finally, no significant correlation was found between studied parameters and subjects’ age.

## Discussion

In this study, we investigated retinal and choroidal vessel features in patients affected by advanced STGD using OCTA. We found that SCP, DCP and CC vessel densities were reduced compared to healthy subjects. In addition, total macular thickness and inner retinal thickness were significantly reduced. Retinal and choriocapillaris vessel densities showed a significant correlation with macular thickness. Macular function assessed using PERG and mfERG was reduced in STDG patients compared to controls, as typically reported in STDG disease [24,25], but were not correlated with anatomical parameters.

In the recent years vascular alterations were investigated in STGD, in particular choroidal alterations were assessed using OCT and retinal angiography [10,12]. A reduction of choriocapillaris thickness and of large and medium size choroidal vessel layer thickness related to the area of outer retinal and RPE atrophy in patients with STGD were demonstrated using OCT [10,26]. Giani et al [11] using ICG reported hypocyanescence from the areas of atrophy more frequent in STGD compared with atrophic AMD, suggesting a possible selective damage of the choriocapillaris in STGD.

These results confirm that choroidal vascular alterations are involved in the pathogenesis of STGD.

Choriocapillaris atrophy was suggested to be related to RPE degeneration due to increased production and accumulation of A2E and related bisretinoids within RPE cells [27,28]. Careful in vivo image analysis, however, does not support this conclusion [8,9].

Choriocapillaris atrophy in turn was hypothesized to cause photoreceptor degeneration [2,6,7].

Choroidal atrophy is an important pathogenic aspect also involved in geographic atrophy.

Similarly, to STGD, advanced dry AMD was reported to be associated with abnormalities in choroidal circulation with histologic evidence of injury of both choriocapillaris and the larger choroidal vessels [29,30]. Choroidal thinning and reduction of choriocapillaris density were also demonstrated by means of OCT and OCTA respectively in dry AMD [31–33].

The results of our study showing significant reduction of CC density were in accordance with OCT studies of STGD and with OCTA studies of dry AMD sharing some similarities with Stargardt disease [29–34].

Features of superficial and deep retinal vascular flow was a novel aspect recently being increasingly investigated in retinal vascular and degenerative diseases by means of OCTA. A reduction of superficial and deep vascular plexuses density was recently demonstrated by means of OCTA in dry AMD patients and in patients with hereditary retinal dystrophies such as retinitis pigmentosa [17,35].

In this study, we found a significant decrease of both SCP and DCP in patients with advanced STGD compared to normal eyes. These modifications were correlated with a significant reduction of total retinal macular thickness and inner retinal thickness.

It is not yet clear whether the reduced vascular density observed in patients with STGD is cause of the disease, or secondary to atrophy of the retinal outer and inner layers. In the first case the vascular depletion could have a causative role in parallel with RPE degeneration. Nevertheless, due to the advanced stage of the disease, a hypothesis of a pathogenic role of reduced vascular density in STDG was not feasible. This aspect would require a study with patients with STDG in an earlier stage than those included in this study.

On the other hand, the vessel density reduction could be related to modification of VEGF concentrations due to RPE degeneration [36]. A previous study in animal model reported capillary rarefaction due to VEGF-signalling inhibition [37]. In the third hypothesis, a reduced metabolic demand due to retinal atrophy would result in a reduced blood supply. This hypothesis would explain the correlation between macular vessel density and retinal atrophy. The greater the retinal atrophy, the lower the vessel density in the area of retinal atrophy.

One major limit of this study was related to possible segmentation error occurring with macular anatomical abnormalities such as macular atrophy typically occurring in STGD and to vessel density analysis errors due to projection artefacts from inner vascular plexus in the outer retina [38]. Nevertheless, reproducibility of OCTA measurements was demonstrated in normal and diseased eyes [16,18,21]. Moreover, in this study an ultimate subjective evaluation of segmentation was made and poor quality images were eliminated.

In conclusion, OCTA has proved to be a useful tool for investigating morphological features of retinal and choroidal vascular plexus in patients with STGD, otherwise not well detectable with other imaging studies. These findings correlated with retinal thickness and macular function examined using electrophysiological tests.
